# Chlorogenic Acid Entrapped in Hybrid Materials with High PEG Content: A Strategy to Obtain Antioxidant Functionalized Biomaterials?

**DOI:** 10.3390/ma12010148

**Published:** 2019-01-04

**Authors:** Michelina Catauro, Federico Barrino, Giovanni Dal Poggetto, Giuseppina Crescente, Simona Piccolella, Severina Pacifico

**Affiliations:** 1 Department of Engineering, University of Campania “Luigi Vanvitelli”, Via Roma 29, I-81031 Aversa, Italy; federicobarrino92@hotmail.it; 2 Ecoricerche, Srl, Via Principi Normanni, 81043 Capua (CE), Italy; ecoricerchesrl@virgilio.it; 3 Department Environmental, Biological and Pharmaceutical Sciences and Technologies, University of Campania “Luigi Vanvitelli”, Via Vivaldi 43, 81100 Caserta, Italy; giuseppina.crescente@unicampania.it (G.C.); simona.piccolella@unicampania.it (S.P.); severina.pacifico@unicampania.it (S.P.)

**Keywords:** organic–inorganic hybrids, sol–gel technique, chlorogenic acid, antioxidant biomaterials, cytotoxicity, antibacterial properties

## Abstract

The formation of pro-oxidant species after implantation of biomaterials could be responsible for the failure of the implant itself, because of oxidative stress-induced damage. In this work, the SiO_2_/polyethylene glycol (PEG)/chlorogenic acid (CGA) hybrids synthesized by the sol–gel method with 50 wt% of the polymer and different amounts of CGA (5, 10, 15 and 20 wt%) were studied. The hybrids soaked in simulated body fluid (SBF) showed the formation of hydroxyapatite layers on their surface, suggesting that the hybrids are bioactive. Their radical scavenging capacity towards DPPH^·^ and ABTS^·+^ (2,2′-Azino-bis(3-ethylbenzthiazoline-6-sulfonic acid), evaluated at three different doses (0.5, 1 and 2 mg), showed probe- and dose-dependent behavior. In addition, the antioxidant properties of CGA were not affected by the presence of high amounts of the polymer. The in vitro biocompatibility in three cell lines (NIH 3T3, HaCaT and SH-SY5Y) was assessed by using the 3-(4,5-dimethyl-2-thiazolyl)-2,5-diphenyl-2H-tetrazolium bromide (MTT) assay. Apart from SH-SY5Y, the cell viability—expressed as mitochondrial redox activity percentage of cells directly exposed to powders—and morphology was not affected, suggesting that the hybrids have the ability to interfere and act selectively against tumor cells. The antibacterial properties of the different materials against *Escherichia coli* and *Enterococcus faecalis* were affected by different amounts of the natural antioxidant component.

## 1. Introduction

Under physiological conditions within the human body, cells are able to counteract the production of free radicals thanks to various enzymes that manage the redox homeostasis. However, when the biological repairing mechanisms are not sufficient and/or pro-oxidant species are overproduced, the homeostatic processes fail and the pro-oxidative/antioxidative cellular balance is compromised. In this condition, called ‘oxidative stress’, reactive oxygen species (ROS), nitrogen species (RNS) and consequently lipid peroxidation (LPO) products promote cellular injury and tissue damage [1,2].

Oxidative stress represents one of the main factors that negatively affect the tissue response to implantation of biomaterials, in that they are able to stimulate the formation of pro-oxidant species, contributing to the occurrence of chronic inflammation. The direct consequence is the loss of the biomaterial’s long-term biocompatibility and function because of the enrolment of leukocytes and macrophages [3]. Thus, the success in biomaterial implantation needs antioxidant supplementation in order to lower oxidative stress-induced damage, which balances the expression of both oxidant production and elimination. However, the bioavailability of antioxidant compounds (e.g., natural phenols and polyphenols), when orally administered, can be affected by several factors that limit their use [4]. In this context, the design of biocompatible materials, able to control oxidant species in situ and lessen all the derived pathological consequences, represents a promising research field. The synthesis of antioxidant polymer-based materials can follow different strategies, varying from the simple encapsulation of antioxidant compounds to the incorporation of antioxidant molecules into the backbone structure through covalent bonds, to form intrinsically antioxidant polymers [5].

The sol–gel route has been widely employed in the preparation of biomaterials, in which organic and inorganic phases are mixed at a molecular level [6,7]. We have used this technique to synthesize organic–inorganic hybrids with the chemical incorporation of natural antioxidant compounds. The flavonol quercetin was the first antioxidant entrapped within a silica-based inorganic material. In particular, several biomaterials, differing in the drug content and in the absence/presence of polymers, such as poly(ε-caprolactone) (PCL) and polyethylene glycol (PEG). Here, we evaluated their influence on structure, biological and antioxidant performances [8,9,10,11,12].

More recently, we have synthesized new phenol-based materials varying the percentage of embedded CGA (5, 10, 15 and 20 wt%) in pure silica and silica-based hybrid materials, containing a high percentage of PEG (50 wt%). The rationale of the choice of CGA relies on the knowledge that this molecule possesses several healthy properties among the antioxidant activity, demonstrated both in vitro and in vivo [13]. The synthesized materials were chemically characterized by Fourier transform infrared (FTIR) spectroscopy and their thermal behavior was evaluated by thermogravimetry under both air and inert N_2_ flowing gas atmospheres [14].

The aim of the present work was the sol–gel synthesis and the biological characterization of the hybrid materials. In order to study the behavior as antioxidant biomaterials of the SiO_2_/PEG/CGA hybrids with different ratios between inorganic and organic composition, the bioactivity and radical scavenging capacity towards DPPH^·^ and ABTS^·+^ were evaluated. Furthermore, the cytotoxicity of the materials, measured by the MTT (3-(4,5-dimethyl-2-thiazolyl)-2,5-diphenyl-2H-tetrazolium bromide) assay—as mitochondrial dehydrogenase activity of the murine fibroblast NIH-3T3, human keratinocyte HaCaT and neuroblastoma SH-SY5Y cell lines—and the antibacterial activity were assessed in function of the different amounts of CGA in the SiO_2_/PEG hybrids.

## 2. Materials and Methods

### 2.1. Sol–Gel Synthesis of Hybrid Materials

The different hybrids were synthesized by the sol–gel method according to a recent study [14]. The inorganic matrix was obtained by means of a solution of the tetraethyl orthosilicate (TEOS, reagent grade, 98%, Sigma Aldrich, Darmstadt, Germany), used as a precursor of silica, ethanol 99% (Sigma Aldrich, Darmstadt, Germany), nitric acid (solution 65%, 89 Sigma Aldrich, Germany), to favor the kinetics of hydrolysis and condensation reactions of the precursor and water. The CGA (≥95%) was acquired from Sigma-Aldrich (Buchs, Switzerland).

**Figure 1:**
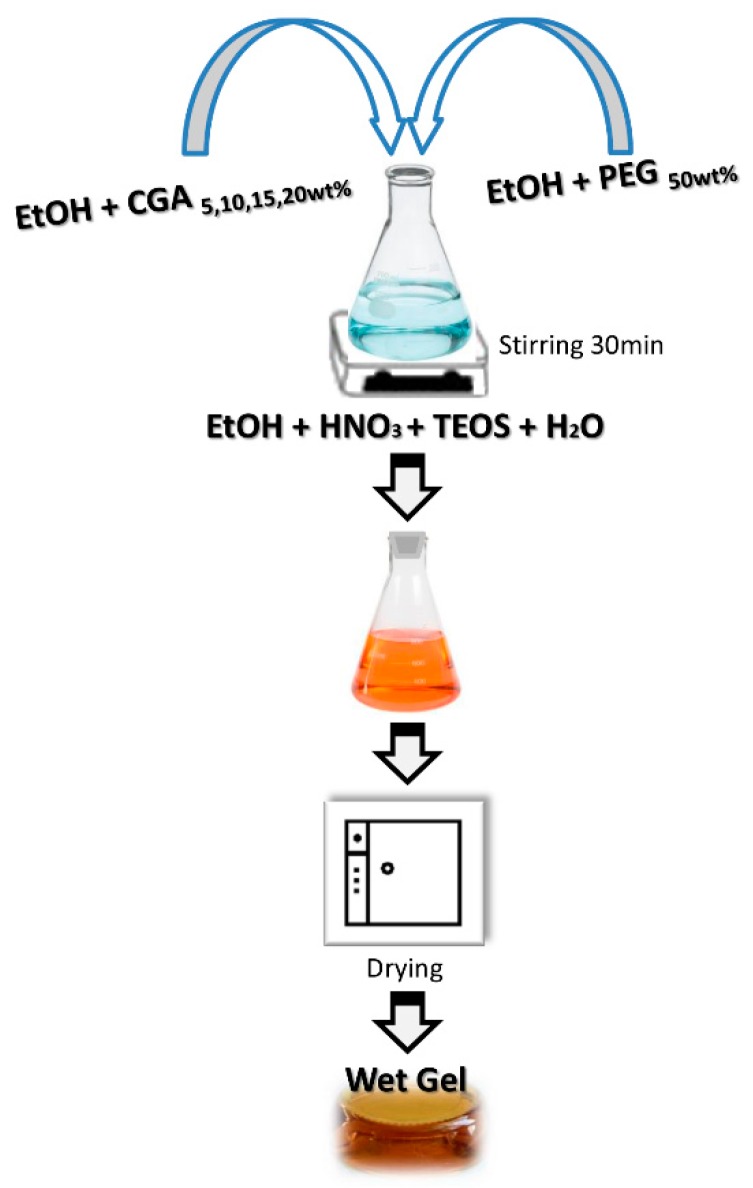
Flow chart of sol–gel synthesis.

PEG (50 wt%) and CGA of different percentages (5, 10, 15, 20 wt%) were dissolved in ethanol and added to the silica matrix (Figure 1). In the solution, the molar ratios between the reagents were: EtOH/TEOS = 6.2, TEOS/HNO_3_ = 1.7 and H_2_O/TEOS = 6.

Afterward, to remove residue solvent, the obtained gel materials were placed in the oven at 40 °C for 24 h to obtain dry powders.

### 2.2. UV–Vis Spectra

The UV–Vis spectra of extracts from hybrids were recorded. To this purpose, powders of synthesized materials (100.0 mg) underwent solid–liquid extraction through ultrasound-assisted maceration (Advantage Plus model ES, Darmstadt, Germany). The extraction was carried out for 1 h using a hydroalcoholic solution (EtOH:H_2_O, 1:1, *v*:*v*; 2.0 mL) as extractant. After centrifugation at 4500 rpm for 5 min, collected supernatants were dried by a rotary evaporator and reconstituted in pure EtOH (1.0 mg/mL). UV–Vis spectra were acquired in the range 200–600 nm by a Shimadzu UV-1700 double beam spectrophotometer (Kyoto, Japan).

### 2.3. Bioactivity Test

The bioactivity of the different materials was evaluated using the simulated body fluid (SBF) solution [15], in which the ion concentration is nearly equal to that in human blood plasma. The formation of hydroxyapatite on the material’s surface was observed by Quanta 200 SEM (FEI, Eindhoven, the Netherlands), equipped with an energy-dispersive X-ray (EDX), after soaking in SBF for 21 days at 37 °C. The reaction of hydroxyapatite nucleation is affected by the total surface area of the material exposed to SBF and its volume; thus, a constant ratio was maintained, according to the literature [15,16,17]. Also, apatite layers on the hybrid materials were analyzed by XRD analysis using a Philips 139 diffractometer, equipped with a PW 1830 generator, a tungsten lamp and a Cu anode, where the source 140 of the X-ray is given by the Cu-Kα radiation (λ = 0.15418 nm).

### 2.4. Antiradical Capacity Assessment

DPPH and ABTS direct contact tests were used to evaluate the radical scavenging capacity of synthesized materials (0.5, 1.0, and 2.0 mg). Briefly, a methanolic solution of DPPH (9.4 × 10^−5^ M; 1.0 mL final volume) was added directly to the powders of hybrid materials. The samples were thoroughly stirred and allowed to react for 30 min at 25 °C. Then, absorbance at 515 nm was measured in reference to a blank, using a Perkin-Elmer Victor3 multi-label reader. Analogously, an ABTS radical cation solution in PBS (pH 7.4; 1.0 mL final volume) was in direct contact with the materials for 6 min, after which the absorbance at 734 nm was measured, versus a blank. The results were expressed in terms of the percentage decrease of the initial DPPH or ABTS^+^ absorption by the test samples [8].

### 2.5. Cell Culture and Cytotoxicity Assessment

Cytotoxicity was measured via the MTT cell viability assay using the HaCat human keratinocyte, NIH-3T3 murine fibroblast and SH-SY5Y human neuroblastoma cell lines. All the cell lines were grown in Dulbecco’s Modified Eagle Medium supplemented with 10% fetal bovine serum, 50.0 U/mL penicillin, and 100.0 μg/mL streptomycin, at 37 °C in a humidified atmosphere containing 5% CO_2_. The powders of each synthesized material (0.5, 1.0 and 2.0 mg) were placed in 12-well plates and the cells were seeded (3.5 × 10^5^ cells/well). After 48 h of incubation, cells were treated with MTT (500 μL; 0.50 mg/mL), previously dissolved in culture media, for 2 h at 37 °C in a 5% CO_2_ humidified atmosphere. The MTT solution was then removed and DMSO was added to dissolve the original formazan. Finally, the absorbance at 570 nm of each well was determined using a Victor3 Perkin Elmer fluorescence and absorbance reader. The cell viability was expressed as a percentage of mitochondrial redox activity of the cells directly exposed to powders, compared to an unexposed control [10].

### 2.6. Antibacterial Activity

*Escherichia coli*, Gram-negative, (ATCC 25922) and *Enterococcus faecalis*, Gram-positive (ATCC 29212), were used to study antibacterial properties of SiO_2_/PEG/CGA hybrids in function of different amounts of CGA [18]. 

The bacterial culture was diluted in distilled water to produce a bacterial cell suspension of 10×10^5^ CFU/mL. *E. coli* was inoculated in TBX Medium (Tryptone Bile X-Gluc) (Liofilchem, Roseto degli Abruzzi (Te), Italy), while, *E. faecalis* was inoculated in Slanetz Bartley Agar Base (Liofilchem, Italy) in presence of 100 mg of the hybrid powders. Afterward, the bacteria were incubated with the materials for 24 h at 44 °C and 48 h at 36 °C, respectively. The microbial growth was evaluated by observing the diameter of the inhibition halo (ID). The obtained values are the mean standard (SD) deviation of the measurements carried out on samples analyzed three times.

## 3. Results

Recent advances in the synthetic methodologies of materials used in the biomedical field see the polymers as main actors, because of their great proficiency to adapt to several specific applications, due to a wide range of physical, chemical and biological properties [19]. Among synthetic polymers used for many decades in the design of biomaterials with controlled structure and dynamic functionality, PEG plays a prominent role. It is known as a versatile, biocompatible polymer, mainly used in polymer-based materials for drug delivery, and is responsible for the improvement of a number of biological properties of drugs [20]. With this in mind and with the aim to propose a new biomaterial with antioxidant activity, able to counteract oxidative stress involved in the pathogenesis of several diseases, we synthesized SiO_2_/PEG/CGA organic–inorganic hybrids, in which the polymer constituted 50 wt% and the natural antioxidant compound was embedded at four different percentages (5, 10, 15 and 20 wt%).

### 3.1. UV–Vis Spectra

**Figure 2:**
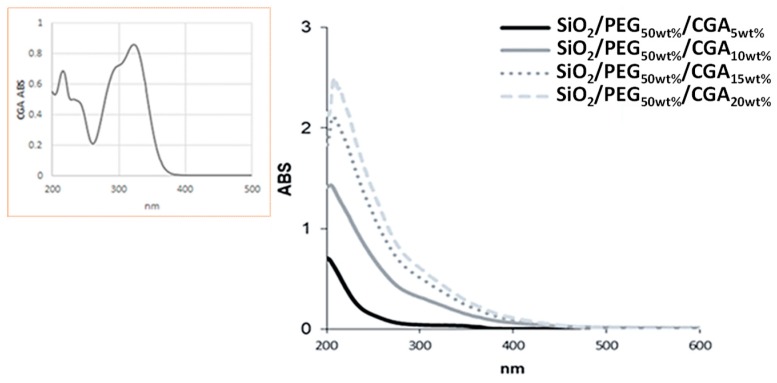
UV–Vis spectra of synthesized hybrids, compared with that of pure chlorogenic acid (in the yellow box).

In order to evaluate the incorporation of CGA in the SiO_2_/PEG-based matrix, the synthesized hybrids were subjected to ultrasound-assisted extraction in a hydroalcoholic solution, and the obtained extracts were investigated by UV–Vis spectroscopy. All the UV–Vis spectra showed a hypsochromic shift of the characteristic bands of CGA, probably due to the occurrence of structural modifications. These results allowed us to deduce that CGA was securely trapped inside the matrix (Figure 2).

The formation of the hydroxyapatite layer on the surfaces of all the hybrid materials was evaluated by SEM images, after soaking for 21 days in simulated body fluid (SBF).

**Figure 3:**
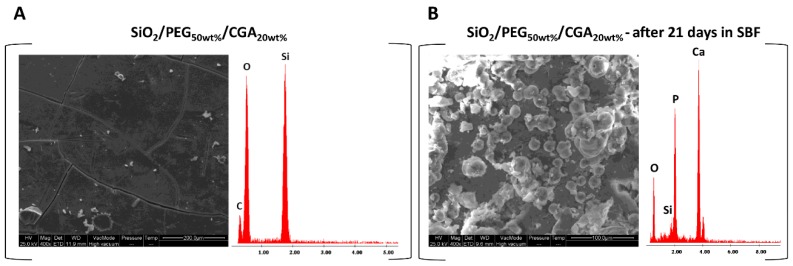
(**A**) Representative SEM micrographs of SiO_2_/PEG_50wt%_/CGA_20wt%_ hybrid and EDX analysis before exposure to simulated body fluid (SBF); (**B**) a representative SEM micrographs of SiO_2_/PEG_50wt%_/CGA_20wt%_ hybrid and EDX analysis after 21 days in SBF.

Comparing the surface materials in which different percentages of CGA were incorporated, no difference was observed in the distribution and amount of hydroxyapatite. Figure 3A shows a representative SEM micrograph of the hybrid with a high percentage of CGA, before soaking in SBF. Homogeneousness and absence of phase separation were visible as well, as in the other materials. Instead, the surface of the materials were covered when the hybrids were soaked in SBF, covered by a precipitate with a globular shape typical of hydroxyapatite (Figure 3B) [15]. In fact, when the materials were soaked in SBF, the interaction between the OH-groups of the silica matrix and the Ca^2+^ ions present in the fluid occurred, leading to the increase of positive surface charge. The Ca^2+^ ions combined with the negative charge of the phosphate ions to form an amorphous phosphate, which spontaneously transformed into hydroxyapatite [Ca_10_(PO_4_)_6_(OH)_2_] [21].

In the energy-dispersive X-ray (EDX) microanalysis of the hybrids after exposure to SBF, the atomic ratio between Ca and P is equal to 1.67, depending on the number of days of exposure. While the EDX of the same samples without exposure to SBF were observed only in the peaks of the elements that formed the hybrids.

**Figure 4:**
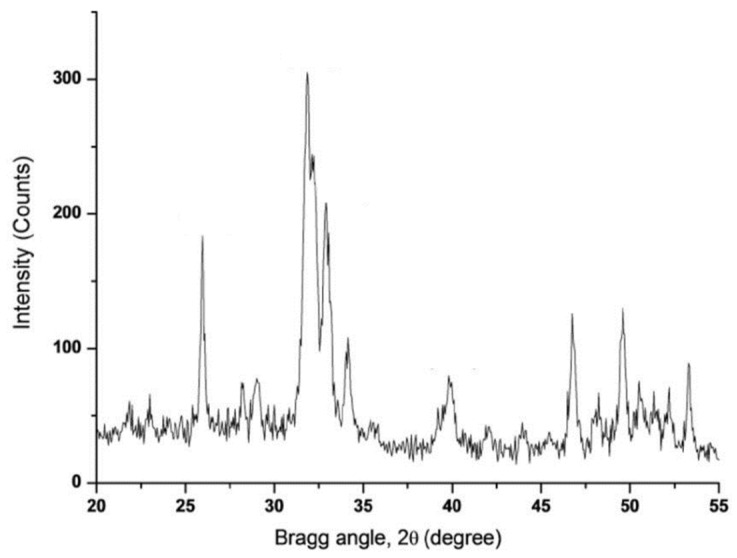
A representative XRD of SiO_2_/PEG_50wt%_/CGA_20wt%_ hybrid soaked in SBF solution for 21 days.

Furthermore, the crystalline hydroxyapatite was confirmed by the intense peaks in the XRD spectrum (Figure 4). In addition, the XRD results suggested that the hydroxyapatite layer on the material’s surface was very thick.

### 3.2. Antiradical Capacity Assessment

**Figure 5:**
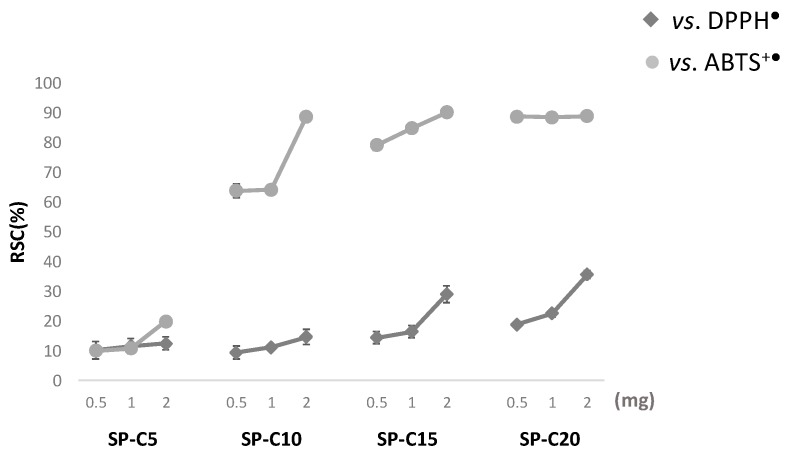
Radical scavenging capacity (RSC, %) versus DPPH (2,2-diphenyl-1-picrylhydrazyl) e ABTS^+^ (2,2’-azino-bis(3-ethylbenzothiazoline-6-sulphonic acid) radicals of synthesized SiO_2_/PEG-based hybrids. Values are the mean ± SD of measurements carried out on three samples (*n* = 3), analyzed three times. SP-C5 = SiO_2_/PEG50%/CGA5%; SP-C10 = SiO_2_/PEG50%/CGA10%; SP-C15 = SiO_2_/PEG50%/CGA15%; SP-C20 = SiO_2_/PEG50%/CGA20%.

The widespread and easy to use DPPH^·^ and ABTS^·+^ methods are useful to evaluate the radical scavenging capacity of a tested sample, providing valid, accurate and reliable data strictly related to the sample chemical features. The adopted methods, which are based on the neutralization reaction induced by the transfer of an electron (ET) and/or a hydrogen atom (HAT), are commonly used in structure–antioxidant activity relationships, which chemically depends on the presence and/or the position of active groups in investigated samples. As CGA easily reacts with both the probes in a dose-dependent manner, their use was also used to verify the CGA preservation during hybrid synthesis (supporting data acquired through characterization techniques)—or at least the maintenance of the CGA antioxidant efficacy by the conservation of those functional groups—which are commonly held responsible for the activity of the natural substance. [22]. Indeed, the synthesized hybrid materials exerted a dissimilar radical scavenging effect towards the two considered probes, but depended on the percentage of CGA (Figure 5). In fact, except for the sample containing the lowest percentage of CGA (SiO_2_/PEG_50wt%_/CGA_5wt%_), which did not show any activity towards both radicals, the other hybrids exerted a more pronounced antiradical capacity versus the radical cation ABTS. Furthermore, for each considered material, the increasing activity appeared directly correlated with the increase in the tested dose (0.5, 1 and 2 mg). An exception to this general trend was represented by the sample having the highest content of CGA (SiO_2_/PEG_50wt%_/CGA_20wt%_). The effectiveness towards ABTS^+^, in this case, reached a maximum—even at the 0.5 mg exposure dose—and remained constant. This showed that the effectiveness in reducing the radical probe of this hybrid is a function of the high percentage of CGA contained therein. The mechanism of chlorogenic acid in exerting free radical scavenging activity, and in particular against DPPH, has been studied by López-Giraldo et al. [23]. The authors demonstrated that the global mechanism of DPPH stabilization likely proceeded ET and involved the dissociation of the phenolic hydroxyl group, the electron transfer from the phenolate anion to DPPH, and finally the stabilization of the DPPH anion by a proton present in the medium.

### 3.3. Cell Culture and Cytotoxicity Assessment

The MTT test was applied to evaluate if the synthesized materials had effects on cell proliferation or showed direct cytotoxic effects, thus affecting biocompatibility. This test determines the activity levels of mitochondrial dehydrogenases toward 3-(4,5-dimethyl-2-thiazolyl)-2,5-diphenyl-2H-tetrazolium bromide (MTT), which is converted to formazan crystals only by living cells [24]. Cell viability was expressed as the mitochondrial redox activity percentage of cells directly exposed to powders, compared to an unexposed control.

NIH-3T3 murine fibroblast, HaCat human keratinocyte and SH-SY5Y human neuroblastoma cell lines were screened. Fibroblasts and keratinocytes are suitable for the use in biocompatibility tests; fibroblasts are representative of the predominant tissue in the body and, in particular, the NIH/3T3 cell line is one of the most frequently used lines in material/cell interaction research [25]. Furthermore, the SH-SY5Y cell line was chosen for their particular sensitivity to oxidative stress onset, making them a model cell system for studying the mechanisms involved in oxidative stress-mediated apoptosis [26].

**Figure 6:**
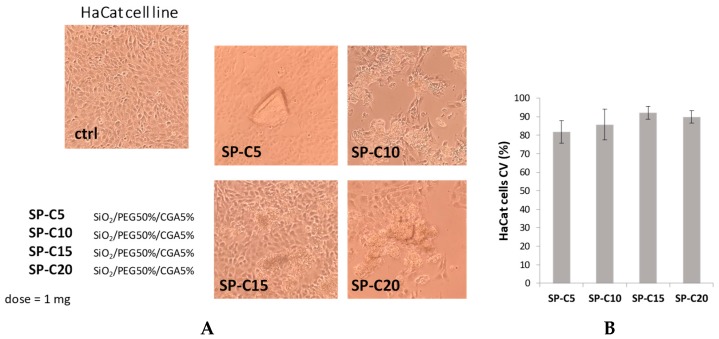
(**A**) Morphological changes of HaCaT cell line treated with synthesized hybrids (1 mg). Representative images were acquired by Inverted Phase Contrast Brightfield Zeiss Primo Vert Microscope. Ctrl = Untreated cells; (**B**) Cell viability (CV, %) of HaCaT cell line.

**Figure 7:**
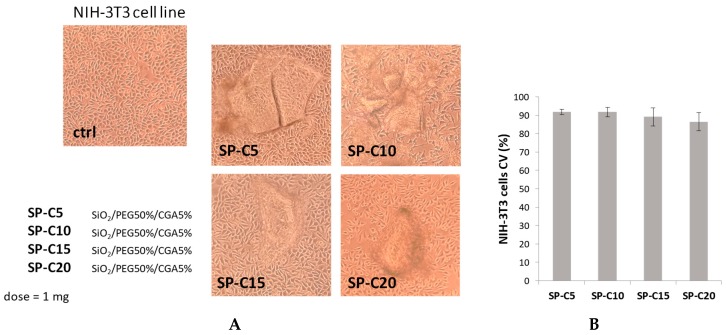
(**A**) Morphological changes of NIH-3T3 cell line treated with synthesized hybrids (1 mg). Representative images were acquired by Inverted Phase Contrast Brightfield Zeiss Primo Vert Microscope. Ctrl = Untreated cells; (**B b**). Cell viability (CV, %) of NIH-3T3 cell line.

These data obtained highlight that the different hybrids do not induce morphological changes and cytotoxic effects on the murine fibroblast and human keratinocyte cell lines tested. In particular, the sample containing a CGA amount equal to 15 wt% induced a slight increase in the percentage of HaCat viable cells, compared to other hybrids (Figure 6), whereas the NIH-3T3 cell viability did not change in response to the different materials used (Figure 7).

**Figure 8:**
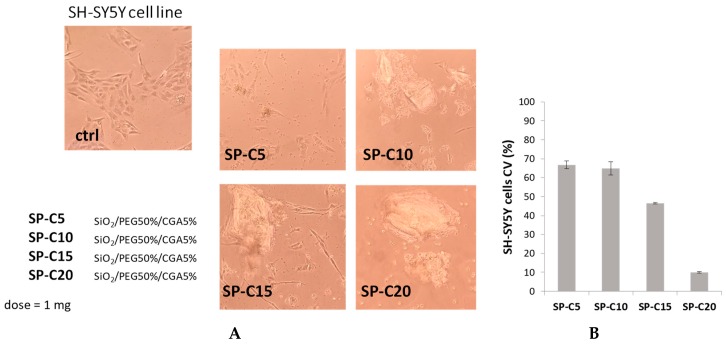
(**A**) Morphological changes of SH-SY5Y cell line treated with synthesized hybrids (1 mg). Representative images were acquired by Inverted Phase Contrast Brightfield Zeiss Primo Vert Microscope. Ctrl = Untreated cells; (**B**) cell viability (CV, %) of SH-SY5Y cell line.

On the contrary, cytotoxic effects were observed for all neuroblastoma cell samples, even when the lowest dose of chlorogenic acid (5 wt%) was entrapped. The cytotoxicity becomes more pronounced with the increase of the CGA amount, reaching about 90% of cell proliferation inhibition at the highest weight percentage (Figure 8). This could be explained taking into account the so-called double-edged sword behavior of phenols and polyphenols [27], meaning that they can be used as antioxidant compounds against oxidative stress at lower doses and as pro-oxidant agents at higher doses. This is in line with our previous findings, which explored the growth inhibitory effect of SiO_2_/PEG/CGA hybrids—with lower amounts of the polymer—against breast cancer and osteosarcoma cell lines [28]. In this context, these encouraging data suggested the ability of the investigated hybrids to induce a consistent variation of protein levels, such as cyclin-dependent kinase inhibitor p21 and caspase-3. Furthermore, the herein observed pro-oxidant effects could depend also on environmental conditions, such as the pH and the presence of transition metals, particularly copper. A viable hypothesis reported in the literature concerns the reduction of Cu(II) to Cu(I), which becomes available in Fenton-like reactions, i.e., promoting the formation of reactive oxygen species [29]. The relationship between phenols and copper was previously investigated in SH-SY5Y cells, treated with oleuropein. The authors demonstrated that depleting cells of copper by treatment with the copper chelator triethylenetetramine, which binds copper with higher affinity than oleuropein, was less toxic than oleuropein with copper-adequate cells [30].

The obtained results allowed us to state that synthesized materials containing different percentages of CGA determined dissimilar responses, based on the considered cell line, and in particular, hybrids are able to interfere and act selectively against tumor cells.

### 3.4. Antibacterial Activity

**Figure 9:**
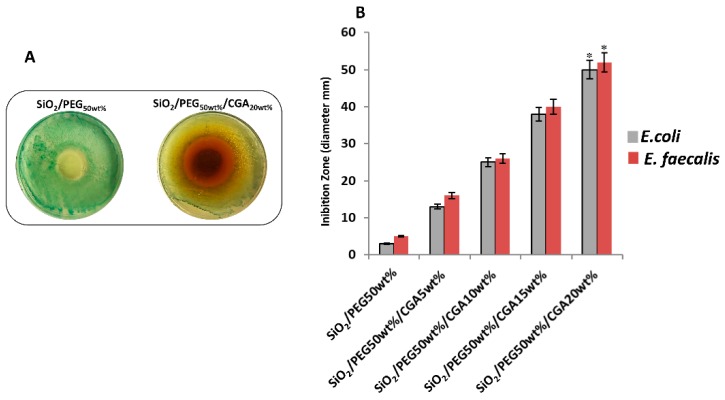
(**A**) Representative inhibition halo (ID) of *E. coli* with SiO_2_/PEG50_wt%_ and SiO_2_/PEG50_wt%_/CGA20_wt%_. (**B**) The diameter (mm) of inhibition zone of all materials incubated with *E. coli* and *E. faecalis*. Values are the mean SD of measurements carried out on samples analyzed three times. The means and S.D. are shown. *, *p* < 0.05 versus the bacteria control treated with hybrids without CGA, or versus the bacteria treated with hybrids containing CGA.

*E. coli* and *E. faecalis* were inoculated in presence of 100 mg of the hybrid powders. Figure 9A shows a representative image of the zones of inhibition that are clearly visible when *E. coli* was incubated with high amounts of CGA, compared with the hybrids containing only PEG. The results of the diameter of the zone of inhibition (Figure 9B) of all the hybrids, suggested that the materials exhibited strong activity against *E. coli* and *E. faecalis* in function of the CGA content, due to disruption the bacterial cell membrane, which lead to cell death [31,32,33]. This happened despite the different cell wall structures of either model bacteria (Gram-positive and Gram-negative). 

The damage of the bacterial cell membrane could be caused by excessive generation of ROS in the cells. In fact, it is known that the equilibrium of levels of ROS is crucial in cells. In particular, it plays many roles in intracellular signaling in different organisms, from bacteria to mammalian cells [34]. Therefore, when ROS increases or depletion occurs, numerous signaling pathways are affected. This leads to malfunction and cellular dysfunction [35].

Lee et al. [36] suggested that CGA-induced bacterial apoptosis-like death is not associated with oxidative stress. Instead, it is possible to observe inhibition of bacterial growth due to ROS depletion by CGA, which is a strong antioxidant compound, as suggested by the ABTS and DPPH results above. Probably, the obtained results are due to CGA inducing ROS depletion in both bacteria, resulting in death from apoptosis.

## 4. Conclusions

The use of antioxidant biomaterials, able to control oxidant species formed in the implantation site, represents a valuable strategy to reduce all the pathological consequences derived from oxidative stress. As a part of a consolidated line of research from our group, in this work we evaluated the capability of new SiO_2_/PEG/CGA hybrids, synthesized by the sol–gel method, to neutralize free radicals, influence cell viability in vitro and inhibit bacterial growth. The radical scavenging effect seemed to be dependent on the amount of CGA and was more pronounced towards ABTS^·+^. The viabilities of the fibroblast and keratinocyte cell lines tested were not compromised. On the contrary, cytotoxicity of the neuroblastoma cell line was observed with increasing doses of CGA. The recorded UV–Vis spectra of all samples, compared with that of pure chlorogenic acid, suggested that there was no release of CGA, which was instead securely trapped inside the SiO_2_/PEG matrix. Finally, the bioactivity was not affected by the presence of CGA in the hybrids, which showed excellent antibacterial activity.

## References

[1] Trachootham D., Lu W., Ogasawara M.A., Valle N.R.-D., Huang P. (2008). Redox regulation of cell survival. Antioxid. Redox Signal..

[2] Rahal A., Kumar A., Singh V., Yadav B., Tiwari R., Chakraborty S., Dhama K. (2014). Oxidative stress, prooxidants, and antioxidants: The interplay. Biomed. Res. Int..

[3] Mouthuy P.-A., Snelling S.J., Dakin S.G., Milković L., Gašparović A.Č., Carr A.J., Žarković N. (2016). Biocompatibility of implantable materials: An oxidative stress viewpoint. Biomaterials.

[4] D’Archivio M., Filesi C., Varì R., Scazzocchio B., Masella R. (2010). Bioavailability of the polyphenols: Status and controversies. Int. J. Mol. Sci..

[5] van Lith R., Ameer G.A. (2016). Antioxidant Polymers as Biomaterial. Oxidative Stress and Biomaterials.

[6] Owens G.J., Singh R.K., Foroutan F., Alqaysi M., Han C.-M., Mahapatra C., Kim H.-W., Knowles J.C. (2016). Sol-gel based materials for biomedical applications. Prog. Mater. Sci..

[7] Catauro M., Pagliuca C., Lisi L., Ruoppolo G. (2002). Synthesis of alkoxide-derived V-Nb catalysts prepared by sol-gel route. Thermochim. Acta.

[8] Catauro M., Papale F., Bollino F., Piccolella S., Marciano S., Nocera P., Pacifico S. (2015). Silica/quercetin sol–gel hybrids as antioxidant dental implant materials. Sci. Technol. Adv. Mater..

[9] Catauro M., Bollino F., Papale F., Piccolella S., Pacifico S. (2016). Sol–gel synthesis and characterization of SiO_2_/PCL hybrid materials containing quercetin as new materials for antioxidant implants. Mater. Sci. Eng. C.

[10] Catauro M., Bollino F., Nocera P., Piccolella S., Pacifico S. (2016). Entrapping quercetin in silica/polyethylene glycol hybrid materials: Chemical characterization and biocompatibility. Mater. Sci. Eng. C.

[11] Nguyen K., Garcia A., Sani M.A., Diaz D., Dubey V., Clayton D., Dal Poggetto G., Cornelius F., Payne R.J., Separovic F. (2018). Interaction of N-terminal peptide analogues of the Na^+^,K^+^-ATPase with membranes. Biochim. Biophys. Acta Biomembr..

[12] Vecchione R., Luciani G., Calcagno V., Jakhmola A., Silvestri B., Guarnieri D., Belli V., Costantini A., Netti P.A. (2016). Multilayered silica-biopolymer nanocapsules with a hydrophobic core and a hydrophilic tunable shell thickness. J. Nanoscale.

[13] Liang N., Kitts D.D. (2015). Role of chlorogenic acids in controlling oxidative and inflammatory stress conditions. Nutrients.

[14] Catauro M., Tranquillo E., Risoluti R., Vecchio Ciprioti S. (2018). Sol-Gel Synthesis, Spectroscopic and Thermal Behavior Study of SiO_2_/PEG Composites Containing Different Amount of Chlorogenic Acid. Polymers.

[15] Kokubo T., Takadama H. (2006). How useful is SBF in predicting in vivo bone bioactivity?. Biomaterials.

[16] Catauro M., Laudisio G., Costantini A., Fresa R., Branda F. (1997). Low Temperature Synthesis, Structure and Bioactivity of 2CaO⋅3SiO_2_ Glass. J. Sol-Gel Sci. Technol..

[17] Catauro M., Dell’Era A., Vecchio Ciprioti S. (2016). Synthesis, structural, spectroscopic and thermoanalytical study of sol-gel derived SiO_2_-CaO-P_2_O_5_ gel and ceramic materials. Thermochim. Acta.

[18] Catauro M., Tranquillo E., Barrino F., Blanco I., Dal Poggetto F., Naviglio D. (2018). Drug Release of Hybrid Materials Containing Fe (II) Citrate Synthesized by Sol-Gel Technique. Materials.

[19] Maitz M.F. (2015). Applications of synthetic polymers in clinical medicine. Biosurf. Biotribol..

[20] D’souza A.A., Shegokar R. (2016). Polyethylene glycol (PEG): A versatile polymer for pharmaceutical applications. Expert Opin. Drug Deliv..

[21] Ohtsuki C., Kokubo T., Yamamuro T. (1992). Mechanism of apatite formation on CaOSiO_2_P_2_O_5_ glasses in a simulated body fluid. J. Non-Cryst. Solids.

[22] Nimse S.B., Pal D. (2015). Free radicals, natural antioxidants, and their reaction mechanisms. RSC Adv..

[23] López-Giraldo L.J., Laguerre M., Lecomte J., Figueroa-Espinoza M.-C., Baréa B., Weiss J., Decker E.A., Villeneuve P. (2009). Kinetic and stoichiometry of the reaction of chlorogenic acid and its alkyl esters against the DPPH radical. J. Agric. Food Chem..

[24] Jaya Seema D., Saifullah B., Selvanayagam M., Gothai S., Hussein M., Subbiah S., Mohd Esa N., Arulselvan P. (2018). Designing of the Anticancer Nanocomposite with Sustained Release Properties by Using Graphene Oxide Nanocarrier with Phenethyl Isothiocyanate as Anticancer Agent. Pharmaceutics.

[25] Rejmontová P., Capáková Z., Mikušová N., Maráková N., Kašpárková V., Lehocký M., Humpolíček P. (2016). Adhesion, proliferation and migration of NIH/3T3 cells on modified polyaniline surfaces. Int. J. Mol. Sci..

[26] Sanvicens N., Gómez-Vicente V., Messeguer A., Cotter T.G. (2006). The radical scavenger CR-6 protects SH-SY5Y neuroblastoma cells from oxidative stress-induced apoptosis: Effect on survival pathways. J. Neurochem..

[27] Castañeda-Arriaga R., Pérez-González A., Reina M., Alvarez-Idaboy J.R., Galano A. (2018). Comprehensive Investigation on the Antioxidant and Pro-Oxidant Effects of Phenolic Compounds: A Double-Edged Sword in the Context of Oxidative Stress?. J. Phys. Chem. B.

[28] Catauro M., Tranquillo E., Salzillo A., Capasso L., Illiano M., Sapio L., Naviglio S. (2018). Silica/Polyethylene Glycol Hybrid Materials Prepared by a Sol-Gel Method and Containing Chlorogenic Acid. Molecules.

[29] Iwasaki Y., Hirasawa T., Maruyama Y., Ishii Y., Ito R., Saito K., Umemura T., Nishikawa A., Nakazawa H. (2011). Effect of interaction between phenolic compounds and copper ion on antioxidant and pro-oxidant activities. Toxicol. In Vitro.

[30] Capo C.R., Pedersen J.Z., Falconi M., Rossi L. (2017). Oleuropein shows copper complexing properties and noxious effect on cultured SH-SY5Y neuroblastoma cells depending on cell copper content. J. Trace Elem. Med. Biol..

[31] Santana-Gálvez J., Cisneros-Zevallos L., Jacobo-Velázquez D.A. (2017). Chlorogenic acid: Recent advances on its dual role as a food additive and a nutraceutical against metabolic syndrome. Molecules.

[32] Lou Z., Wang H., Zhu S., Ma C., Wang Z. (2011). Antibacterial activity and mechanism of action of chlorogenic acid. J. Food Sci..

[33] Li G., Wang X., Xu Y., Zhang B., Xia X. (2014). Antimicrobial effect and mode of action of chlorogenic acid on Staphylococcus aureus. Eur. Food Res. Technol..

[34] Ray P.D., Huang B.-W., Tsuji Y. (2012). Reactive oxygen species (ROS) homeostasis and redox regulation in cellular signaling. Cell Signal..

[35] Zhang J., Wang X., Vikash V., Ye Q., Wu D., Liu Y., Dong W. (2016). ROS and ROS-mediated cellular signaling. Oxid. Med. Cell. Longev..

[36] Lee B., Lee D.G. (2018). Depletion of reactive oxygen species induced by chlorogenic acid triggers apoptosis-like death in Escherichia coli. Free Radic. Res.

